# Young People’s Use of E-Cigarettes across the United Kingdom: Findings from Five Surveys 2015–2017

**DOI:** 10.3390/ijerph14090973

**Published:** 2017-08-29

**Authors:** Linda Bauld, Anne Marie MacKintosh, Brian Eastwood, Allison Ford, Graham Moore, Martin Dockrell, Deborah Arnott, Hazel Cheeseman, Ann McNeill

**Affiliations:** 1 Institute for Social Marketing, University of Stirling, Stirling FK9 4LA, UK; a.m.mackintosh@stir.ac.uk (A.M.M.); a.j.ford@stir.ac.uk (A.F.); 2 UK Centre for Tobacco and Alcohol Studies; 3 Alcohol, Drugs and Tobacco Division, Health Improvement Directorate, Public Health England, Skipton House, 80 London Road, London SE1 6LH, UK; brian.eastwood@phe.gov.uk (B.E.); martin.dockrell@phe.gov.uk (M.D.); 4 Centre for the Development and Evaluation of Complex Interventions for Public Health Improvement (DECIPHer), School of Social Sciences, Cardiff University, 1-3 Museum Place, Cardiff CF10 3BD, UK; mooreg@cardiff.ac.uk; 5 Action on Smoking and Health (ASH); 67-68 Hatton Garden, London EC1N 8JY, UK; deborah.arnott@ash.org.uk (D.A.); hazel.cheeseman@ash.org.uk (H.C.); 6 Addictions Department, Institute of Psychiatry, Psychology and Neuroscience, King’s College London, 4 Windsor Walk, London SE5 8BB, UK; ann.mcneill@kcl.ac.uk

**Keywords:** smoking, tobacco, e-cigarettes, youth, prevalence, surveys

## Abstract

Concern has been expressed about the use of e-cigarettes among young people. Our study reported e-cigarette and tobacco cigarette ever and regular use among 11–16 year olds across the UK. Data came from five large scale surveys with different designs and sampling strategies conducted between 2015 and 2017: The Youth Tobacco Policy Survey; the Schools Health Research Network Wales survey; two Action on Smoking and Health (ASH) Smokefree Great Britain-Youth Surveys; and the Scottish Schools Adolescent Lifestyle and Substance Use Survey. Cumulatively these surveys collected data from over 60,000 young people. For 2015/16 data for 11–16 year olds: ever smoking ranged from 11% to 20%; regular (at least weekly) smoking between 1% and 4%; ever use of e-cigarettes 7% to 18%; regular (at least weekly) use 1% to 3%; among never smokers, ever e-cigarette use ranged from 4% to 10% with regular use between 0.1% and 0.5%; among regular smokers, ever e-cigarette use ranged from 67% to 92% and regular use 7% to 38%. ASH surveys showed a rise in the prevalence of ever use of e-cigarettes from 7% (2016) to 11% (2017) but prevalence of regular use did not change remaining at 1%. In summary, surveys across the UK show a consistent pattern: most e-cigarette experimentation does not turn into regular use, and levels of regular use in young people who have never smoked remain very low.

## 1. Introduction

Considerable concern has been expressed about the use of electronic cigarettes or nicotine vapourisers by young people [1]. Some studies have suggested that these products may serve as a ‘gateway’ to tobacco use, with young people who experiment with e-cigarettes being more likely to go on to try tobacco smoking in longitudinal studies [2]. Tobacco use remains the leading preventable cause of death in the world and preventing youth uptake is an important global public health priority [3]. However, recorded increases in e-cigarette use among the young have coincided with continued declines in youth smoking rates in the UK, USA and in other countries where e-cigarette use has increased [4,5,6]. These two findings seem at odds with each other—If youth e-cigarette use is causing smoking and is being observed in an increasing proportion of young people, why are youth smoking rates continuing to decline?

There are likely to be several explanations for this. Some authors have pointed to the inability of existing longitudinal studies to establish causality, or the small sample sizes within these studies which mean that they do not apply to larger populations of young people [7]. They also argue that these studies are unable to fully account for the fact that young people who experiment with e-cigarettes may be exposed to a wide range of factors, some of them difficult to measure, which make them more susceptible to future tobacco use [5]. Another explanation may lie in the way in which e-cigarette and tobacco use is measured in surveys. In many cases, questions simply ask about ever or recent (past 30 day) use and do not explore regular use. Given that fewer than one in four young people who try tobacco go on to become regular smokers [8,9], it may be that accurate assessment of frequency of both smoking and e-cigarette use is important to better understand population level trends.

In the United Kingdom (England, Wales, Scotland, and Northern Ireland), considerable effort has been invested in tracking youth smoking rates and in recent years questions on e-cigarettes have been added to surveys. The UK is a country of particular interest, as it is has introduced rigorous regulations to reduce the potential risks of e-cigarettes but has sought to maximise the opportunity that e-cigarettes present for smoking cessation and smokefree homes. It is therefore integral to such a strategy that any adverse impacts on adolescents are monitored in a country which seeks to balance health risks and opportunities. The questions added to youth surveys in the UK assess both ever and more frequent use of both tobacco products and e-cigarettes [10]. Most publications have focused on single surveys conducted at the UK level, or in one of the four countries which make up the UK [11,12,13]. The current study builds on a previous analysis that provided an initial comparative overview of UK youth survey data on e-cigarette use from 2014 [10]. The current study includes the latest data from across the UK and provides a more in-depth analysis, aiming to assess recent trends in ever and regular use of tobacco and electronic cigarettes. Comparable data from four surveys conducted in 2015–2016 are included, supplemented by very recent additional data from one survey (covering all of the UK with the exception of Northern Ireland) in 2017.

## 2. Materials and Methods

### 2.1. Design

Data came from five surveys conducted between 2015 and 2017: the Youth Tobacco Policy Survey (YTPS); the Schools Health Research Network (SHRN) Wales survey; two Action on Smoking and Health (ASH) Smokefree GB-Youth Surveys; and the Scottish Schools Adolescent Lifestyle and Substance Use Survey (SALSUS). The surveys differed in both administration method and approach to measuring e-cigarette use.

YTPS data came from Wave 8 of the UK Youth Tobacco Policy Survey (YTPS), a long-running, repeat cross-sectional study examining the impact of tobacco policies on adolescents. The survey comprised an in-home face-to-face interview, followed by a self-completion questionnaire to gather more sensitive information on smoking behaviour and e-cigarette use. Both the face-to-face and self-completion questionnaires were completed using Computer Assisted Telephone Interviewing (CAPI). FACTS International (Ashford, UK), a market research company, recruited participants and conducted the fieldwork during August and September 2016. Parental and participant informed consent was obtained prior to each interview. Ethics approval was granted by the School of Health Sciences Research Ethics Committee at the University of Stirling.

ASH Smokefree GB-Youth data came from two waves of annual national internet surveys of 11–18 year olds in Great Britain (England, Wales and Scotland with the exclusion of Northern Ireland), one conducted in March and April 2016 [11] and another in March and April 2017 (currently unpublished). YouGov (a public limited company, London, UK) was commissioned by the charity ASH to conduct annual surveys from 2013 to 2017. Informed consent was provided by either a parent of those aged 11 to 15, or by the individuals aged 16 to 18. For further details on methods see Eastwood et al. [11]. To facilitate comparison with the other surveys, only data from 11 to 16 year olds in the ASH Smokefree GB surveys are included in this paper.

SHRN Wales data came from a schools based survey administered in 87 secondary schools in Wales between September and December 2015. The survey was an online, self-completion survey, available in English and Welsh. Schools managed its implementation using their own facilities. For further details on the methods see de Lacy et al. [13]. The survey was voluntary and completed anonymously. Parents were provided with information in advance of the survey and were given the option of withdrawing their child from the survey. Ethical approval (SREC/1530) was granted by the Cardiff University School of Social Sciences Research Ethics Committee.

SALSUS data came from the 2015 wave of a long-running schools based self-completion survey, administered under exam conditions by teachers in a mixed ability class. For further details on methods see the SALSUS technical report [14]. For the first time, in the 2015 wave, conducted between September 2015 and January 2016, half of the sample completed the survey online while the other half completed it using the traditional paper based approach. Data from the online and paper based responses are merged as the mode effect report [15] indicated no significant difference by mode of completion in key behavioural variables. Parents and pupils were provided with information about the survey and parents were provided with slips to return to the school if they did not wish their child to participate. Pupils were informed that they did not have to complete the questionnaire or they could refuse to answer specific questions.

### 2.2. Sampling Strategy

YTPS: Using random location quota sampling, a sample of 11 to 16 year olds was drawn from households across the UK. Sampling involved a random selection of 92 electoral wards stratified by Government Office Region and A Classification Of Residential Neighbourhoods (ACORN) classification (a geodemographic classification system that describes demographic and lifestyle profiles of small geographic areas) to ensure the coverage of a range of geographic areas and socio-demographic backgrounds. Wards covering the islands, areas north of the Caledonian Canal, or those with fewer than three urban/suburban Enumeration Districts were excluded from the sampling frame for cost and practicality reasons. In each selected ward, interviewers approached households until a quota of 15 interviews was obtained, balanced across gender and age. A total sample of 1213 was achieved.

ASH Smokefree GB-Youth: This survey has been running annually since 2013 and, each year, a proportion of respondents are followed-up and a new cross-sectional sample is also recruited. For the purpose of this paper, data are only presented for newly recruited respondents, aged 11 to 16 years, in each year. A total of 1205 11 to 16 year olds were recruited to the cross-sectional sample in 2016, and 1361 were recruited in 2017.

SHRN Wales: All 113 schools in the Wales School Health Research Network, with representation from all local authority areas [13], were invited to participate in the survey. A total of 87 schools participated. Schools were asked to include all students, but if this was not possible, to include a minimum of two randomly selected, mixed ability classes per year. A total sample of 32,479 was achieved.

SALSUS: A random, nationally representative sample of S2 (second year) and S4 (fourth year) secondary school pupils was selected with classes as the primary sampling unit. All local authority and independent schools in Scotland were eligible for inclusion in the sample, with the exception of special schools. A total of 13,607 S2 and 11,697 S4 pupils responded. S2 pupils are referred to as ‘13 year olds’ and S4 pupils are referred to as ‘15 year olds’ for ease. Some pupils may be slightly older or younger.

### 2.3. Measures

The way in which measures of smoking prevalence and e-cigarette prevalence were assessed in the surveys varied slightly. However, the response categories for each product were sufficiently similar to enable the classification of respondents into ‘never use’, ‘ever use’, and ‘regular (at least weekly) use’. Table 1 outlines the different questions used and how each response category links to ‘never’, ‘ever’, and ‘regular’ e-cigarette use.

#### 2.3.1. E-Cigarette Use

Some of the surveys initially asked about the awareness of e-cigarettes and filtered out those who had not heard of e-cigarettes. Within this paper, the prevalence of e-cigarette use is calculated as a percentage of all respondents, with those who have never heard of e-cigarettes classed as having never used e-cigarettes.

**Figure 1:**
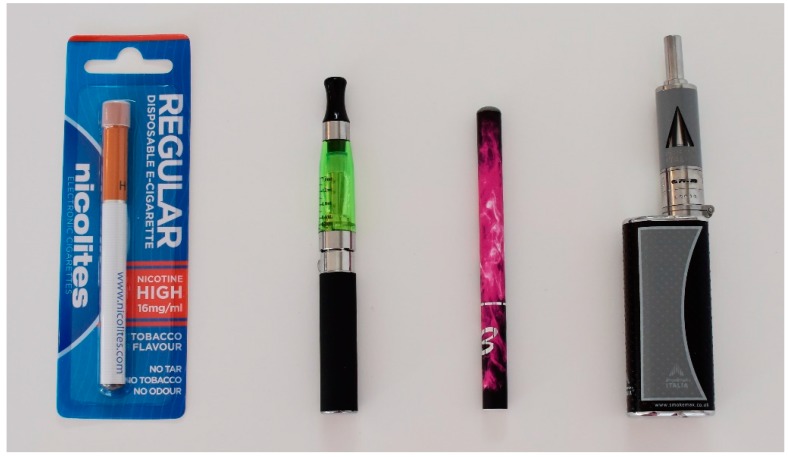
Visual prompt used in the Youth Tobacco Policy Survey (YTPS) to illustrate different styles of e-cigarettes.

YTPS: Questions on e-cigarettes were introduced with: “*Now we’d like you to think about electronic cigarettes, sometimes called e-cigarettes, e-shisha or vaping devices. E-cigarettes puff a vapour that looks like smoke but, unlike normal cigarettes, you don’t light them with a flame and they don’t burn tobacco. Have you ever heard of e-cigarettes?*” Subsequent questions on e-cigarettes were asked of all respondents, regardless of whether they had heard of e-cigarettes, by including a description and visual representation of e-cigarettes: “*E-cigarettes come in different styles. Some look similar to normal cigarettes and have a glowing tip while some look more like pens. Here is a picture of some different styles of e-cigarettes.”* (Figure 1) *“Have you ever seen any of these types of e-cigarettes?”* One item in the self-completion questionnaire assessed e-cigarette use: *“Which of these best describes whether or not you have ever used or tried e-cigarettes?”* Table 1 shows the response options and classification into ‘never use’, ‘ever use’ and ‘regular use’.

ASH Smokefree GB-Youth: Youth were asked “*Have you ever heard of e-cigarettes? They are also sometimes called shisha pens, vapourisers or electronic cigarettes.*” Those who had heard of e-cigarettes were then asked “*Which of the following describes your experience of e-cigarettes?”* Table 1 shows the response options. Those who had never heard of e-cigarettes were treated as having never tried e-cigarettes.

SHRN Wales: E-cigarettes were introduced as “*a device used to inhale vapour, sometimes called vaping, including those which look like a conventional cigarette with a glowing tip, or like a pen or a small bottle (a ‘tank’)*”. Young people were asked at what age they first used an e-cigarette, with options other than “never” classified as ‘ever users’. ‘Ever users’ were also asked “*How often do you use e-cigarettes at present?” with answers of “every day” or “at least once a week but not every day*” classed as ‘regular use’.

SALSUS: E-cigarettes were introduced as “*An electronic cigarette (sometimes called an ‘e-cigarette’) is a tube that can look like a normal cigarette, can have a glowing tip and puffs a vapour that looks like smoke but, unlike normal cigarettes, they don’t burn tobacco*”. Pupils were given a list of response options (see Table 1) and were asked which one best described them.

#### 2.3.2. Smoking Status

YTPS: Smoking status was established based on two questions. Never smokers consisted of participants who indicated “*I have never smoked”* in response to one question, and confirmed ‘*I have never tried smoking, not even a puff or two’* at a subsequent question. All participants who did not classify as *never smokers* were classed as having *ever smoked*. Regular smokers, a subset of ever smokers, consisted of participants who indicated “*I usually smoke between one and six cigarettes per week’* or ‘*I usually smoke more than six cigarettes per week”*.

ASH Smokefree GB-Youth: Smoking status was established based on one question. Youth were categorised into never, ever, and regular smokers as follows: “*I have never smoked cigarettes, not even a puff or two*” (categorised as ‘never smokers’); “*I have only ever tried smoking cigarettes once”*, “*I used to smoke sometimes but I never smoke cigarettes now*”, and “*I sometimes smoke cigarettes now but less than one a week*”, “*I usually smoke between one and six cigarettes a week*” and “*I usually smoke more than six cigarettes a week*” (categorised as ‘ever smokers’). Regular smokers, a subset of ever smokers, comprised of those who smoked at least one cigarette a week.

SHRN Wales: Smoking status was established based on two questions. Never smokers were those who answered ‘*never*’ to “*At what age did you first do the following things: Smoked a cigarette (more than a puff)*”. All participants who did not classify as never smokers were classed as having *ever smoked*. A subsequent question on frequency of current smoking was used to identify regular smokers as those who answered “*At least once a week, but not every day*” or “*Every day”*.

SALSUS: Smoking status was established based on one question. Never smokers consisted of participants who indicated ‘I have never smoked’. All participants who did not classify as *never smokers* were classed as having *ever smoked*. Regular smokers, a subset of ever smokers, consisted of participants who indicated “*I usually smoke between one and six cigarettes a week*’ or ‘*I usually smoke more than six cigarettes a week*”.

### 2.4. Data Analysis

Descriptive data on the prevalence of smoking and e-cigarette use are presented graphically from all the surveys in 2015 and 2016 and then data from the most recent ASH Smokefree GB-Youth survey, conducted in 2017, is presented alongside its 2016 counterpart. E-cigarette prevalence is presented for all respondents, and also for never smokers and regular smokers. Data from the YTPS have been weighted to standardise by age and gender. Data from ASH Smokefree GB-Youth 2016 and 2017 were weighted to be representative of all 11–18 year olds in Great Britain, using age, gender and region of residence. Data from SALSUS were weighted to be representative of pupils across Scotland. For brevity only weighted percentages are presented. For the two waves of the ASH Smokefree GB-Youth survey, the Pearson Chi-square statistic was used to test for independence of ever-use of e-cigarette and year of survey. This statistic was corrected for the stratified survey design with a second-order correction, and converted into an F statistic as per Eastwood et al. [16].

## 3. Results

### 3.1. 2015/2016 Surveys

#### 3.1.1. Smoking Prevalence

**Figure 2:**
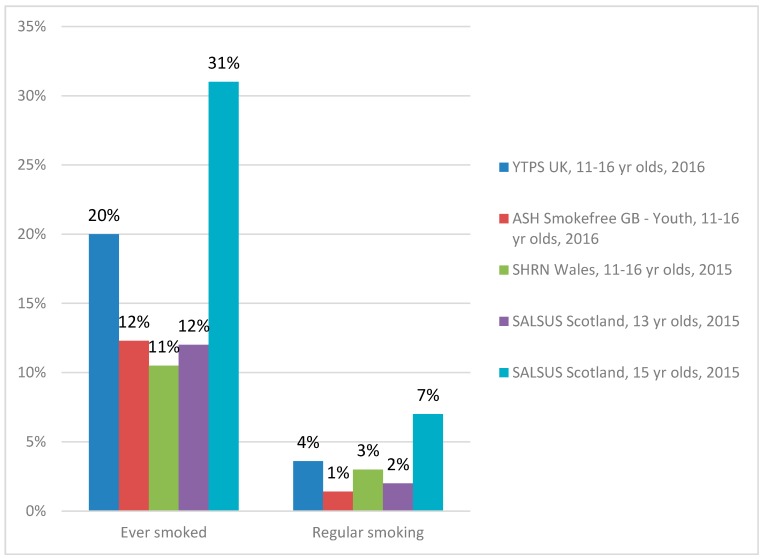
Prevalence of smoking in teenagers, UK surveys 2015/2016. Notes: Youth Tobacco Policy Survey (YTPS), United Kingdom, *n* = 1213 (2016); Action on Smoking and Health Smokefree Great Britain-Youth Survey *n* = 1205 (2016); Schools Health Research Network (SHRN), Wales, *n* = 32,479 (11 to 16 year olds in 2015); and, Scottish Schools Adolescent Lifestyle and Substance Use Survey (SALSUS), *n* = 13,607 (13 year olds in 2015), *n* = 11,697 (15 year olds in 2015).

In the YTPS survey of 11 to 16 year olds, one in five (20%) had ever smoked (including having tried smoking) (Figure 2) while the ASH Smokefree GB-Youth survey and the Wales survey indicated a lower prevalence of ever smoking in the same age group, at 12% and 11% respectively. Among 13 year olds in the SALSUS survey more than one in ten (12%) had ever smoked, rising to almost one in three (31%) by age 15. Regular smoking was low in all the surveys, ranging from between 1% and 4% for 11 to 16 year olds. Among 13 year olds in SALSUS, 2% were regular smokers increasing to 7% by age 15.

#### 3.1.2. Prevalence of E-Cigarette Use

**Figure 3:**
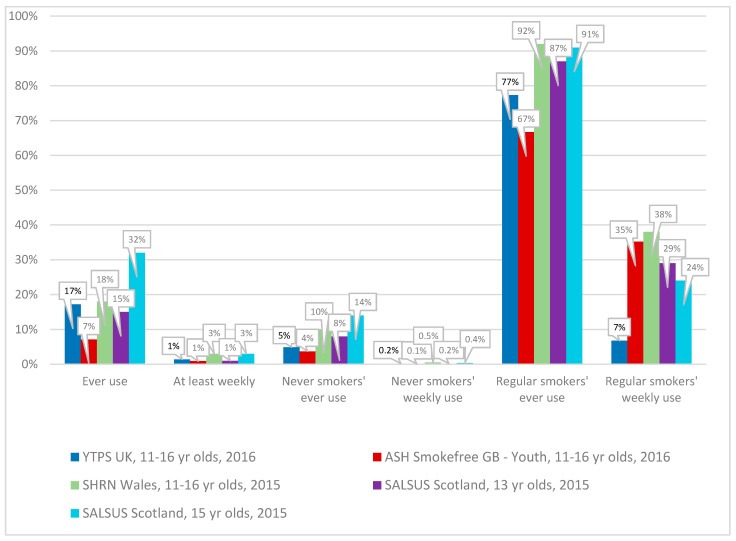
Prevalence of electronic cigarette (e-cigarette) use in teenagers, UK surveys 2015/2016. Notes: Youth Tobacco Policy Survey (YTPS), United Kingdom, *n* = 1213 (2016); Action on Smoking and Health Smokefree Great Britain-Youth Survey *n* = 1205 (2016); Schools Health Research Network (SHRN), Wales, *n* = 32,479 (11 to 16 year olds in 2015); and, Scottish Schools Adolescent Lifestyle and Substance Use Survey (SALSUS), *n* = 13,607 (13 year olds in 2015), *n* = 11,697 (15 year olds in 2015). Base for regular smokers in YTPS and ASH Smokefree GB is less than 50.

Figure 3 presents the prevalence of ‘ever use’ and ‘at least weekly use’ of e-cigarettes firstly from all respondents from each of the surveys conducted in 2015/16 and then separately for never smokers and regular smokers.

##### All Respondents: Ever Use of E-Cigarettes

Among all respondents in each of the surveys, prevalence of ‘ever use’ ranged from 7% to 18% among 11 to 16 year olds. The lowest reported prevalence (7%) was in the ASH Smokefree GB-Youth survey while the YTPS and SHRN Wales surveys reported prevalence among 11 to 16 year olds to be 17% and 18% respectively. The SALSUS school surveys indicated prevalence among 13 year olds to be 15% rising to 32% at age 15.

##### All Respondents: Regular (at Least Weekly) Use of E-Cigarettes

Across the surveys, the prevalence of regular (at least weekly) use, among all respondents, ranged from 1% to 3%. The YTPS, ASH Smokefree GB-Youth and SALSUS survey of 13 year olds all indicated regular use to be 1%, while the SHRN Wales survey of 11 to 16 year olds and the SALSUS survey of 15 year olds indicated 3% regularly used e-cigarettes.

##### Never Smokers: Ever Use of E-Cigarettes

Across the surveys, reports of ‘ever use’ of e-cigarettes, among never smokers, ranged from 4% to 10% among 11 to 16 year olds. The YTPS Survey and ASH Smokefree GB-Youth survey indicated 5% and 4%, respectively, of never smokers had ever used an e-cigarette while, in the SHRN Wales survey, 10% of never smokers had ever used an e-cigarette. The SALSUS survey indicated 8% of never smokers had tried e-cigarettes at age 13 and, by age 15, 14% had tried them.

##### Never Smokers: Regular (at Least Weekly) Use of E-Cigarettes

Among never smokers, the regular use of e-cigarettes was very low in all surveys, ranging from 0.1% to 0.5%.

##### Regular Smokers: Ever Use of E-Cigarettes

In each of the surveys the majority of regular smokers (ranging from 67% of ASH Smokefree GB-Youth to 92% of SHRN Wales 11 to 16 year olds) had tried e-cigarettes.

##### Regular Smokers: Regular (at Least Weekly) Use of E-Cigarettes

Regular (at least weekly) use among regular smokers ranged from 7% to 38% among 11 to 16 year olds. Among the small number of regular smokers (*n* = 44) in the YTPS survey of 11 to 16 year olds, only a small minority (7%, *n* = 3) also used e-cigarettes regularly. Similarly, the ASH Smokefree GB-Youth survey had a very small number of regular smokers (*n* = 14) with just over one in three using e-cigarettes regularly. In the other surveys, regular use among regular smokers ranged from 24% to 38%.

### 3.2. ASH Smokefree GB-Youth 2017

**Figure 4:**
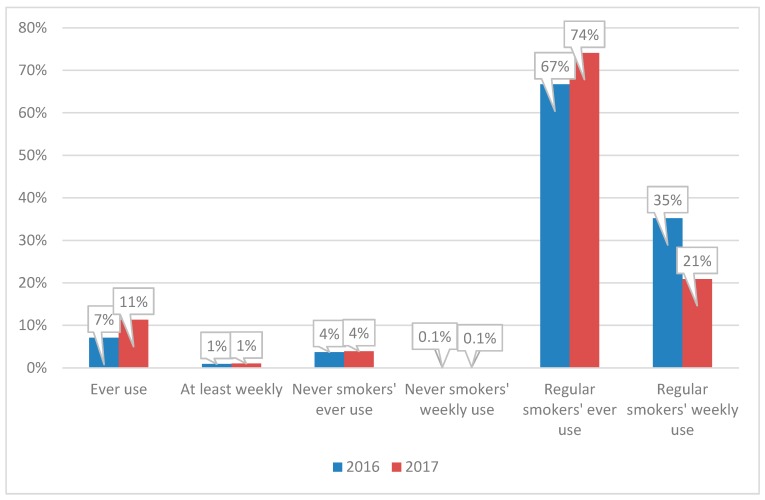
Prevalence of electronic cigarette (e-cigarette) use in 11 to 16 year olds, ASH Smokefree GB-Youth survey, 2016/2017. Notes: ASH Smokefree GB-Youth Survey *n* = 1205 (2016), *n* = 1361 (2017). Base for regular smokers in 2016 survey is only 14 and for 2017 is only 28.

More recent data are available for 2017 from the ASH Smokefree GB-Youth survey only. Overall, ever use of e-cigarettes was reported by 11% of 11 to 16 year olds in 2017, with 1% reporting at least weekly use of e-cigarettes (Figure 4). Ever use of e-cigarettes was reported by 4% of never-smokers, as compared with 74% of regular smokers. Weekly use of e-cigarettes was reported by 21% of regular smokers and 0.1% of never-smokers.

Compared with the 2016 data this indicates a rise in prevalence of ever use from 7% in 2016 to 11% in 2017 (*p* < 0.01). However, prevalence of regular use did not change, remaining very low (1%). Prevalence of ‘ever use’ or ‘regular use’ among never smokers also remained unchanged. The small base sizes of regular smokers in the surveys make it difficult to detect differences between the two years.

## 4. Discussion

Our study reported e-cigarette and tobacco cigarette ever and regular use among 11–16 year olds across the UK. It is important to differentiate between smoking and vaping as these are different behaviours. However, from a public health perspective e-cigarettes are most useful as devices to deter smoking or support cessation and thus in this paper both behaviours have been described. A proactive and pragmatic approach is being taken towards electronic cigarette use in the UK, including promoting them as less harmful nicotine delivery devices to adult smokers who cannot or will not stop smoking, while introducing policies to protect never smoking youth from using e-cigarettes including banning almost all forms of marketing and introducing age of sale laws. Although we found that around a tenth to a fifth of 11–16 year olds report having tried e-cigarettes, only 3% or less report using them at least weekly, most of whom are regular smokers, with less than 0.5% of never smokers reporting weekly e-cigarette use. This pattern was consistent across different surveys from around the UK and suggests that, for now, experimentation with e-cigarettes does not necessarily translate into regular use, particularly among never smokers.

The data are limited by being drawn from surveys with very different designs and involving participants from different combinations of the countries making up the UK. Each of the methodologies used in the surveys have limitations. For example, schools-based surveys are more likely to miss those adolescents who are most at risk of taking up smoking. Internet-based samples may also not be fully representative of the general population, but weighting the sample reduces the risk of bias. Bias may also be introduced as participation of some adolescents was conditional on parental consent. The different methodologies have strengths however, for example online surveys can provide data much more quickly and hence some estimates reported here are very recent. Nevertheless, the consistency of the overall finding (regular e-cigarette use being largely confined to regular smokers) despite these different methodologies gives us confidence in our findings. The very low regular use of e-cigarettes and tobacco cigarettes overall mean that the numbers are often too small to make sub-group comparisons between surveys or over time, however, these low numbers are indicative of the fact that both regular smoking and regular e-cigarette use are rare among youth in the UK.

Our findings indicate that there is no evidence of e-cigarettes driving smoking prevalence upwards. This is important, and suggests that fears about e-cigarettes as a gateway to more youth becoming smokers are not currently justified, at least in the UK. In Scotland, regular smoking has declined markedly since the turn of the century when it was just under 30% for 15 year olds and around 10% for 13 year olds, as compared with 7% among 15 year olds and 2% among 13 year olds in 2015 [6]. In England, a national schools survey in 2014 [17] found that 18% of 11 to 15 year olds reported trying smoking at least once, the lowest level recorded since the survey began in 1982, continuing a decline from 42% who reported trying smoking observed in 2003. Similarly, 3% of pupils reported smoking at least weekly, again a decline since 2002, when 10% were at least weekly smokers. These findings are consistent with ours reported here for Great Britain/UK. The English schools survey also found that 8% of 15 year olds reported smoking at least weekly which is similar to the 7% of pupils aged 15 years old reporting regular weekly smoking in Scotland that we report here. Nevertheless, experimentation with tobacco smoking needs to be further reduced, and new tobacco control measures, such as reducing the accessibility of cheap tobacco to adolescents through reducing the number of outlets selling tobacco or raising the age of legal sale of tobacco to 21, should be given serious consideration [18].

Our findings on e-cigarettes indicate that while a significant minority of adolescents report experimenting with e-cigarettes, most experimentation does not become regular use. This suggests that the dependence potential of e-cigarettes might be lower than for tobacco cigarettes where the trajectory from ever use to regular weekly use appears higher [19,20]. Experimentation is considerably lower among never smokers and regular use remains very low (less than 0.5%) among never smokers. The SALSUS data presented here can be compared with the previous published survey data from Scotland, which suggests an increase in experimentation with e-cigarettes between 2013 and 2015 and an increase in regular e-cigarette use among smokers. Similarly in Wales, experimentation has increased from 12% (2013) to 18% (2015) and regular use from 1.5% (2013) to 3% (2015). However, the more recent data from the ASH Smokefree GB-Youth survey provided here suggest regular use may now have plateaued. The English national schools survey in 2014 [17] found that 22% of pupils reported some experimentation with e-cigarettes which is consistent with estimates taken from our more recent surveys and trends in the ASH youth data reported elsewhere [11].

There is some variation in estimates of ever smoking between surveys in our study. This may reflect different age groups (particularly the higher overall rates in the SALSUS data for 15 year olds only). Other reasons may be due to different survey administration methods (school-based, online and household surveys) and extent of geographical coverage across the UK. However, the estimates of regular smoking are more consistent at around 3–4%.

Our data on youth who have ever tried e-cigarettes is comparable with the international data. A review of youth e-cigarette studies published between January 2014 and January 2016 found that similar rates of young people had ever tried an e-cigarette in Canada, Greece, Finland, New Zealand and Ireland, with proportions of between 14.6% and 24% [21]. Recently published data from the 2014 US National Youth Tobacco Survey (NYTS) similarly reported ever use among middle and high school students as 19.4% [22]. Furthermore, as with our data, youth e-cigarette experimentation in other countries has followed an upward trend in recent years [21].

International comparisons on regular e-cigarette use are more challenging due to differences in survey measures assessing frequency of use. In Greenhill et al.’s [21] review of all existing youth studies, only three studies from outside the UK were identified as assessing some form of regular use. Differences in the measures, however, make direct comparisons with our data impossible. More commonly assessed in youth surveys is use of e-cigarettes in the past 30 days. Across countries rates of 30-day e-cigarette use are considerably lower than for ever-use [21,22,23]. Youth who report past-30 day use are consistently more likely to also report use of tobacco products. For example, in the NYTS, 87% of the 9.3% who reported any past 30-day e-cigarette use had ever used a tobacco product and the proportion of never smokers using e-cigarettes frequently, i.e., on 10 days or more in the past 30 days, was less than 0.1% [22]. This is consistent with our data where regular-use of e-cigarettes among never smokers is rare. An important priority for future research is to agree a common set of core measures for youth surveys internationally, similar to recent recommendations for surveys of adults [24].

## 5. Conclusions

This paper highlights the current rates of e-cigarette use among youth in the UK, where e-cigarettes form a part of a tobacco harm reduction policy landscape. Whilst it is estimated that there are 2.9 million e-cigarette current users among adults in Great Britain [25], regular use among 11–16 year olds remains very low, at 3% or less, and remains largely confined to regular smokers. Regular e-cigarette use among never smokers is very rare. These low rates of regular use suggest that youth experimentation is not currently leading to greater frequency of use, however, comparing youth e-cigarette data and trends across surveys and countries is crucial to better understand youth trends. Survey measures must be designed to assess frequency of use, rather than just ever or past 30 day use [24].

## Figures and Tables

**Table 1:** Measures of e-cigarette use from the different surveys.

Use		YTPS	SHRN Wales	ASH Smokefree GB–Youth #	SALSUS
**Never use**		I have never used e-cigarettes	At what age did you first do the following things? (If there is something that you have never done choose the “never” category) …used an e-cigarette.	I have never used an e-cigarette	I have never used an e-cigarette
			never		
**Ever use**		I have only ever tried e-cigarettes once or twice I have used e-cigarettes in the past, but I never use them now I occasionally use e-cigarettes (less than once a month) I use e-cigarettes at least once a month	any response other than never	I have only tried an e-cigarette once or twice I use e-cigarettes sometimes but no more than once a month I use e-cigarettes more than once a month but less than once a week	I used to use e-cigarettes but don’t use them anymore I have tried an e-cigarette once I have tried e-cigarettes a few times I use e-cigarettes sometimes, but no more than once a month
	Regular use *	I use e-cigarettes at least once a week	How often do you use e-cigarettes at present? Every day At least once a week but not every day	I use e-cigarettes more than once a week but not every day	I use e-cigarettes once a week or more
				I use e-cigarettes every day	

# ASH Smokefree GB-Youth: only asked of those who’d heard of e-cigarettes. If they hadn’t heard of e-cigarettes they were considered de facto ‘Never users’. * ‘Regular use’ is a sub-category of ‘ever use’. YTPS: UK Youth Tobacco Policy Survey; SHRN Wales: Wales School Health Research Network; ASH Smokefree GB-Youth: Action on Smoking and Health Smokefree Great Britain-Youth; SALSUS: Scottish Schools Adolescent Lifestyle and Substance Use Survey.
